# YOLO-V5 based deep learning approach for tooth detection and segmentation on pediatric panoramic radiographs in mixed dentition

**DOI:** 10.1186/s12880-024-01338-w

**Published:** 2024-07-11

**Authors:** Busra Beser, Tugba Reis, Merve Nur Berber, Edanur Topaloglu, Esra Gungor, Münevver Coruh Kılıc, Sacide Duman, Özer Çelik, Alican Kuran, Ibrahim Sevki Bayrakdar

**Affiliations:** 1 Department of Orthodontics, Faculty of Dentistry, Recep Tayyip Erdogan University, Rize, Turkey; 2 Pedodontics, Private Practice, Trabzon, Turkey; 3 Department of Oral and Maxillofacial Radiology, Faculty of Dentistry, Inonu University, Malatya, Turkey; 4 Department of Pedodontics, Faculty of Dentistry, Inonu University, Malatya, Turkey; 5 Department of Pedodontics, Faculty of Dentistry, Beykent University, Istanbul, Turkey; 6 Department of Mathematics-Computer, Faculty of Science, Eskisehir Osmangazi University, Eskisehir, Turkey; 7 Department of Oral and Maxillofacial Radiology, Faculty of Dentistry, Kocaeli University, İzmit, Kocaeli 41190 Turkey; 8 Department of Oral and Maxillofacial Radiology, Faculty of Dentistry, Eskisehir Osmangazi University, Eskişehir, Turkey

**Keywords:** Artificial intelligence, Deep learning, Tooth enumeration, Panoramic radiographs, Pediatric dentistry

## Abstract

**Objectives:**

In the interpretation of panoramic radiographs (PRs), the identification and numbering of teeth is an important part of the correct diagnosis. This study evaluates the effectiveness of YOLO-v5 in the automatic detection, segmentation, and numbering of deciduous and permanent teeth in mixed dentition pediatric patients based on PRs.

**Methods:**

A total of 3854 mixed pediatric patients PRs were labelled for deciduous and permanent teeth using the CranioCatch labeling program. The dataset was divided into three subsets: training (*n* = 3093, 80% of the total), validation (*n* = 387, 10% of the total) and test (*n* = 385, 10% of the total). An artificial intelligence (AI) algorithm using YOLO-v5 models were developed.

**Results:**

The sensitivity, precision, F-1 score, and mean average precision-0.5 (mAP-0.5) values were 0.99, 0.99, 0.99, and 0.98 respectively, to teeth detection. The sensitivity, precision, F-1 score, and mAP-0.5 values were 0.98, 0.98, 0.98, and 0.98, respectively, to teeth segmentation.

**Conclusions:**

YOLO-v5 based models can have the potential to detect and enable the accurate segmentation of deciduous and permanent teeth using PRs of pediatric patients with mixed dentition.

**Supplementary Information:**

The online version contains supplementary material available at 10.1186/s12880-024-01338-w.

## Introduction

Panoramic radiographs (PRs) are an essential tool for the diagnosis and treatment of patients in cases where a thorough clinical examination is not sufficient [1]. Pediatric dentists widely use PRs as essential diagnostic tools, enabling them to observe anatomical structures and carefully differentiate between deciduous and permanent teeth, dental restorations, and pathological conditions [2]. Furthermore, PRs are a suitable imaging technique for children due to their low radiation dose, technical efficiency, and simplicity [3]. 

In the interpretation of PRs, the identification and numbering of teeth is an important part of the correct diagnosis. This is especially important during the mixed dentition period, when both erupted and unerupted teeth are present. Correct identification facilitates the diagnostic process, but misidentification can result in the scheduling of unnecessary appointments and the administration of inappropriate treatments. In addition, the manual process of identifying and numbering erupted and unerupted teeth is time-consuming and depends on the qualifications of the dentist examining the radiograph [4]. Consequently, the automation of these processes is a significant concern. Nevertheless, the automation of tooth detection and segmentation can be regarded as the initial and most challenging stage in the development of artificial intelligence (AI) systems that are capable of interpreting images and distinguishing pathologies from anatomical structures [4]. The challenge arises from the large number of teeth in each jaw during tooth segmentation, the close proximity of neighboring teeth, variations in tooth density, and the various looks of teeth at different phases of development [5]. Because of intrinsic constraints, implementing these processes in panoramic radiography becomes increasingly difficult [6]. Within this framework, the identification and segmentation of dental structures in panoramic radiographs pose a greater challenge compared to the segmentation of bone structures. Therefore, it is crucial to ensure the highest level of accuracy in this initial phase of artificial intelligence system development [4]. 

AI has become increasingly popular in radiographic interpretation, including in dentomaxillofacial radiology. AI-based methods assist in image interpretation, providing faster data identification and improved diagnostic accuracy [7]. This is particularly beneficial in recognizing and managing dental and craniofacial conditions, while also eliminating errors associated with human fatigue [4]. The Deep Learning (DL) technique utilizes convolutional neural networks (CNN), a type of DL architecture, to automatically learn from datasets [8]. This learning process results in the creation of a learning model that is built upon large volumes of data rather than relying on instructions. There are numerous studies in the literature combine DL with maxillofacial radiography [9]. DL algorithms have been studied for detecting, classifying, or diagnosing diseases or anatomical structures in dentomaxillofacial radiology [10].

You Only Look Once (YOLO) algorithm, which is one of the deep learning algorithms, is one of the most popular CNNs for object segmentation and detection. As the name suggests, the YOLO algorithm can detect objects in a single pass, providing fast and high-accuracy object detection and segmentation [11]. While other CNNs can also be used for object detection, YOLO is the latest version of object recognition models [12]. The initial version of this deep learning algorithm, YOLO-v1, had a fully connected output layer, supporting only the full input resolution during the testing phase [13]. YOLO-v2 was developed to address the shortcomings of YOLO-v1 and has the capability to detect up to 9000 objects, achieving more accurate results [14]. YOLO-v3, compared to its predecessors, introduces changes in the structure of the model, providing flexibility in terms of speed and accuracy [15]. YOLO-v4, aiming to overcome the limitations of previous versions, strives to find the best balance between input network resolution, convolutional layer count, parameter count, and layer outputs [16]. YOLO-v5 is a more practical and powerful object detection model compared to other versions [17]. Unlike previous models written in the C programming language, YOLO-v5 is written in Python and operates in Pytorch. This makes it more accessible for installation and integration with Internet of Things devices. Additionally, YOLO-v5 has the capability to achieve successful detections in a significantly shorter time compared to other models. When compared to the Darknet library of YOLOv4, the Pytorch library of YOLOv5 is more extensive, indicating a greater potential for contributions and future growth [18]. 

Although there are studies on tooth segmentation and detection in PRs of various patient groups in the literature [4, 19–22], there are very few publications in the mixed dentition period in pediatric patients and there is no study using the YOLO-V5 DL model in this patient group. This study evaluates the effectiveness of YOLO-V5, a DL method, in the automatic detection, segmentation and numbering of primary and permanent teeth in mixed dentition pediatric patients based on PRs.

## Materials and methods

In this study, Checklist for Artificial Intelligence in Medical Imaging (CLAIM) and Standards for the Reporting of Diagnostic Accuracy Studies (STARD) Checklist were followed for preparing the manuscript. The non-interventional Clinical Research Ethical Committee of Eskisehir Osmangazi University approved the study protocol (decision no. 04.10.2022/22). The study was conducted in accordance with the principles of the Declaration of Helsinki. Since the patients included in the study were children, informed consent and signatures were obtained from their legal responsible persons regarding the use of their PRs.

### Patient selection

In this retrospective observational study, 3854 anonymous PRs from a pediatric patient population aged 5–13 years obtained from the archive of the Department of Oral and Maxillofacial Radiology, Eskisehir Osmangazi University were evaluated. PRs that contained artefacts related to superimposed metal, positioning errors or motion were excluded from the dataset. The study included PRs exhibiting teeth with dental caries, restorative fillings, developmental anomalies, rotated teeth, and supernumerary teeth. There were no gender or ethnicity discrepancies observed, and the data was anonymized before being uploaded into the labelling system.

### Radiographic data set

The PRs were acquired using the Planmeca Promax 2D (Planmeca, Helsinki, Finland) panoramic dental imaging unit. The imaging parameters used were 68 kVp, 16 mA, and 13 s.

### Ground truth labelling

Labelling is the process of identifying areas in an image and determining which region the object belongs to. The labelling process was conducted by a team of experts, including two orthodontists (B.B. and M.N.B.), two pediatric dentists (T.R. and E.G.), and two oral and maxillofacial radiologists (E.T. and A.K.). They employed the web-based CranioCatch annotation software (Eskişehir, Turkey) to complete the task. Once all the annotation procedures had been completed, the labellings were checked by senior oral and maxillofacial radiologist (I.S.B.) and senior pediatric dentists (S.D. and M.C.K.) with at least 10 years of experience. Full agreement was achieved on all labels.

The FDI tooth numbering system was used to identify deciduous and permanent teeth in the PR during the labelling process. The outer boundaries of these anatomical areas were defined using polygonal segmentation and saved in JSON (JavaScript Object Notation) format, following a meticulous delineation procedure.

### Deep convolutional neural network

**Fig. 1:**
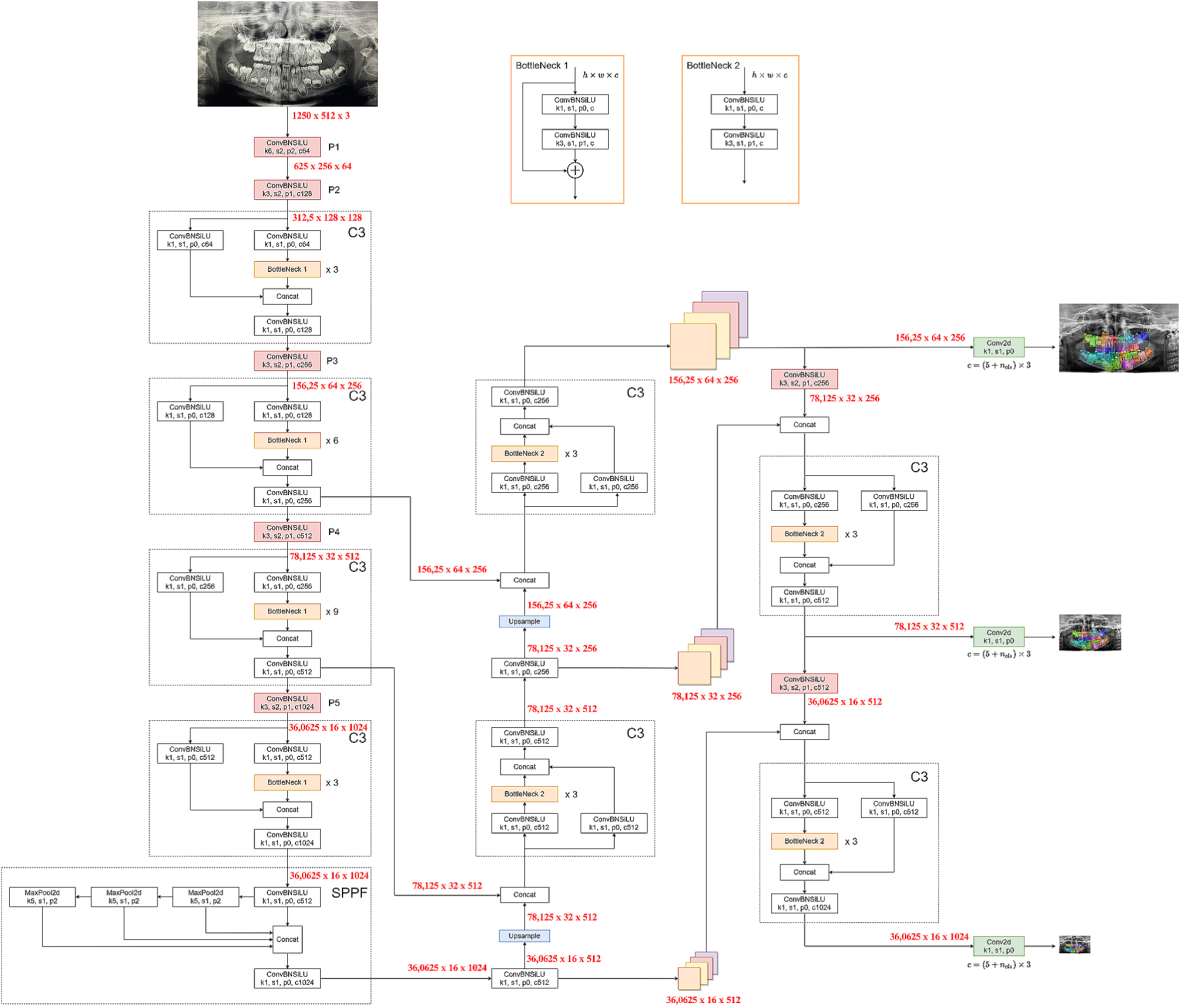
Diagram showing the architecture of the developed YOLOv5 model

The YOLO-v5 algorithm establishes superiority over its predecessor models through techniques such as the Cross Stage Partial Networks backbone, PANet neck, Focus module, FPN module, SAM module, CBAM module, GIOU loss, CIOU loss, and DIOU loss. The YOLOv5 algorithm has five different types: YOLOv5s, YOLOv5n, YOLOv5m, YOLOv5l, and YOLOv5x. YOLOv5x, referred to as “xlarge,” is larger than the other models and, although slower, its success in achieving a higher accuracy rate is attributed to its 88.8 million parameters. In this study, the YOLO-v5x model has been developed separately for both detection and segmentation tasks. The fundamental architecture of YOLOv5x consists of Backbone, Neck, and Head components (Fig. 1).

- Backbone: The YOLOv5x backbone consists of 106 layers and is based on the CSPDarknet53 architecture. The backbone of YOLOv5x comprises 53 ResNet blocks, each consisting of three 3 × 3 convolutional layers, totaling 23.8 million parameters.
- Neck: The neck of YOLOv5x is based on the PANet architecture, which is a combination of FPN and PAN. PANet enhances object detection by merging feature maps of different dimensions. The neck of YOLOv5x consists of 12 PANet blocks, each containing two 3 × 3 convolutional layers. The neck of YOLOv5x encompasses a total of 3.5 million parameters.
- Head: The head of YOLOv5x consists of 6 YOLO layers, with each YOLO layer comprising three 3 × 3 convolutional layers. YOLOv5x’s head contains a total of 10.4 million parameters.

YOLOv5x is characterized by a set of hyperparameters that exert considerable influence over its performance. These hyperparameters intricately regulate the model’s behaviour during both the training and inference phases, underscoring their critical significance in the attainment of optimal outcomes.

Hyperparameters used for augmentation:

- hsv_s: 0.7.
- hsv_v: 0.4.
- translate: 0.1.
- scale: 0.5.
- mosaic: 0.0.
- mixup: 0.0.
- copy_paste: 0.0.
- flipud: 0.0.
- fliplr: 0.0.

### Developing of tooth detection and segmentation models

#### Pre-processing

**Fig. 2:**
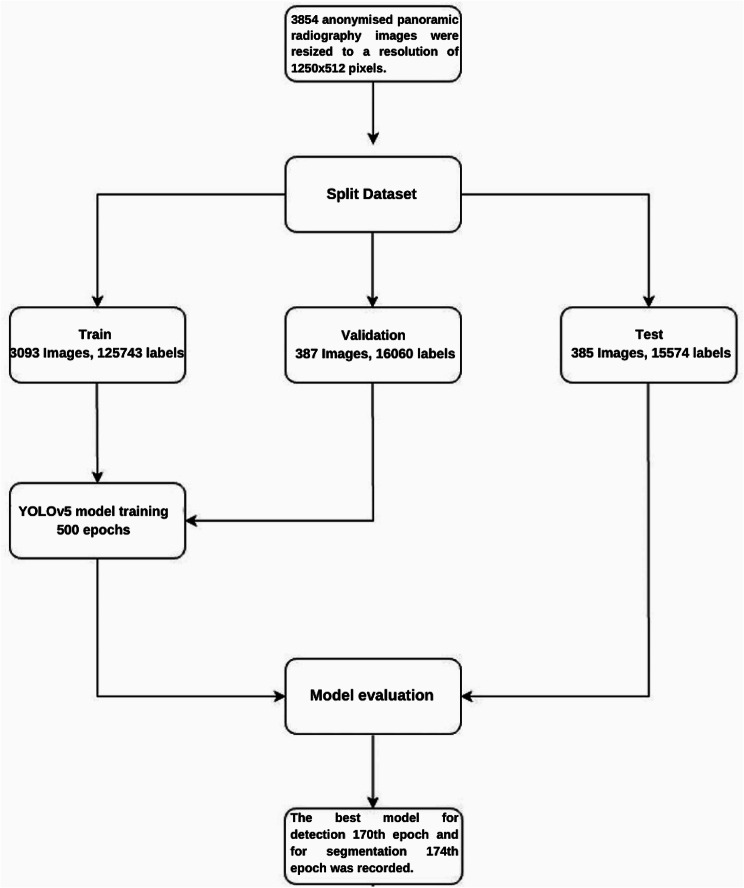
The diagram of tooth detection and segmentation model (CranioCatch, Eskisehir-Turkey)

For the development of the tooth detection and segmentation model, 3854 anonymized, PR images were resized to 1280 × 512 pixels. To maintain the integrity of the evaluation process, we partitioned the dataset into three distinct subsets. 80% of the dataset was designated for training purposes, while 10% was reserved for validation to fine-tune parameters. The remaining 10% was used for final model testing (Fig. 2).

- *Training data*: 80% of the images (3093 Images, 125,743 labels).
- *Validation data*: 10% of the images (387 Images, 16,060 labels).
- *Test data*: 10% of the images (385 Images, 15,574 labels).

### Training

Following the establishment of YOLO models, an AI algorithm was developed using Python, an open-source programming language (version 3.6.1; Python Software Foundation, Wilmington, DE, USA). The PyTorch module used the YOLO-v5x network to create the AI algorithm. The training approach was implemented using Eskişehir Osmangazi University Faculty of Dentistry Dental-AI Laboratory’s computer technology (Appendix A). The training and validation datasets were used for planning and estimating the ideal weight values for the CNN algorithm. The model’s hyperparameters were set based on variables such as the volume and type of data, the number of classes, the diversity of the classes, and the overall success rate. For PRs, the augmentation hyperparameters for tooth detection and segmentation were calibrated based on the size of the dataset. Flipud and Fliplr hyperparameters were set to flipud = 0.0 and fliplr = 0.0 to prevent the model from being misinterpreted by the numbering on the right and left sides in accordance with the PR data type. Because the PRs resolution was thought to be sufficient, img_size was set to 1280. In order to effectively analyse a sizable amount of data at once and take into consideration the graphics card’s computational capacity, a batch size of eight was selected. The learning rate was reduced in order to maintain a high success rate. The class number parameter was set to 52, given the presence of 52 distinct classes. Stochastic Gradient Descent (SGD) optimization algorithm, prominently employed in YOLOv5, served as the chosen optimization technique. SGD is acknowledged for its simplicity and effectiveness in refining model parameters. The foundational principle of SGD involves the computation of the gradient, representing the derivative of the loss function, and subsequently updating the model’s weights based on this gradient. According to PR numbering data diversity and data type, the number of anchors was determined to be a 4.0. The model was trained over 500 epochs using the YOLOv5x architecture, with a learning rate of 0.01. The best model for teeth detection 170th epoch and the best model for teeth segmentation 174th epoch was recorded.

### Metrics of models performance

The effectiveness of the models was assessed by means of a confusion matrix, which is a visual representation of the difference between predicted and actual outcomes. Each of the detection and segmentation models was evaluated by calculating performance metrics based on the interaction of True Positive (TP: accurate diagnoses correctly detected/segmented), False Positive (FP: diagnoses mistakenly identified and imprecisely detected/segmented), and False Negative (FN: diagnoses incorrectly detected/segmented) evaluations. This evaluative framework encompassed metrics such as the sensitivity, precision, performance (F1 score), and mean average precision-0.5 (mAP-0.5) for each model.

## Results

**Fig. 3:**
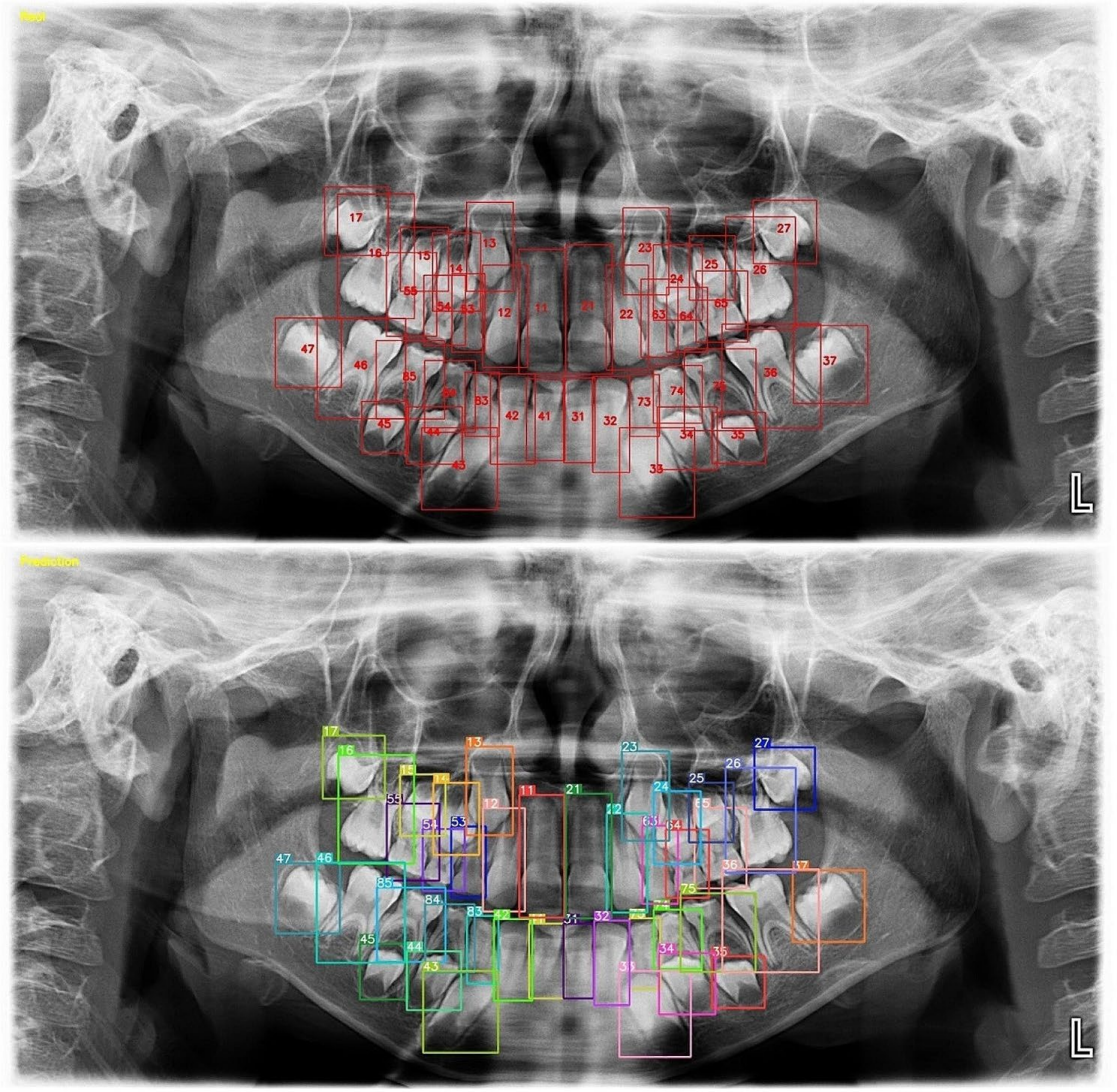
Tooth detection in PRs of pediatric patients with mixed dentition using an AI model

**Fig. 4:**
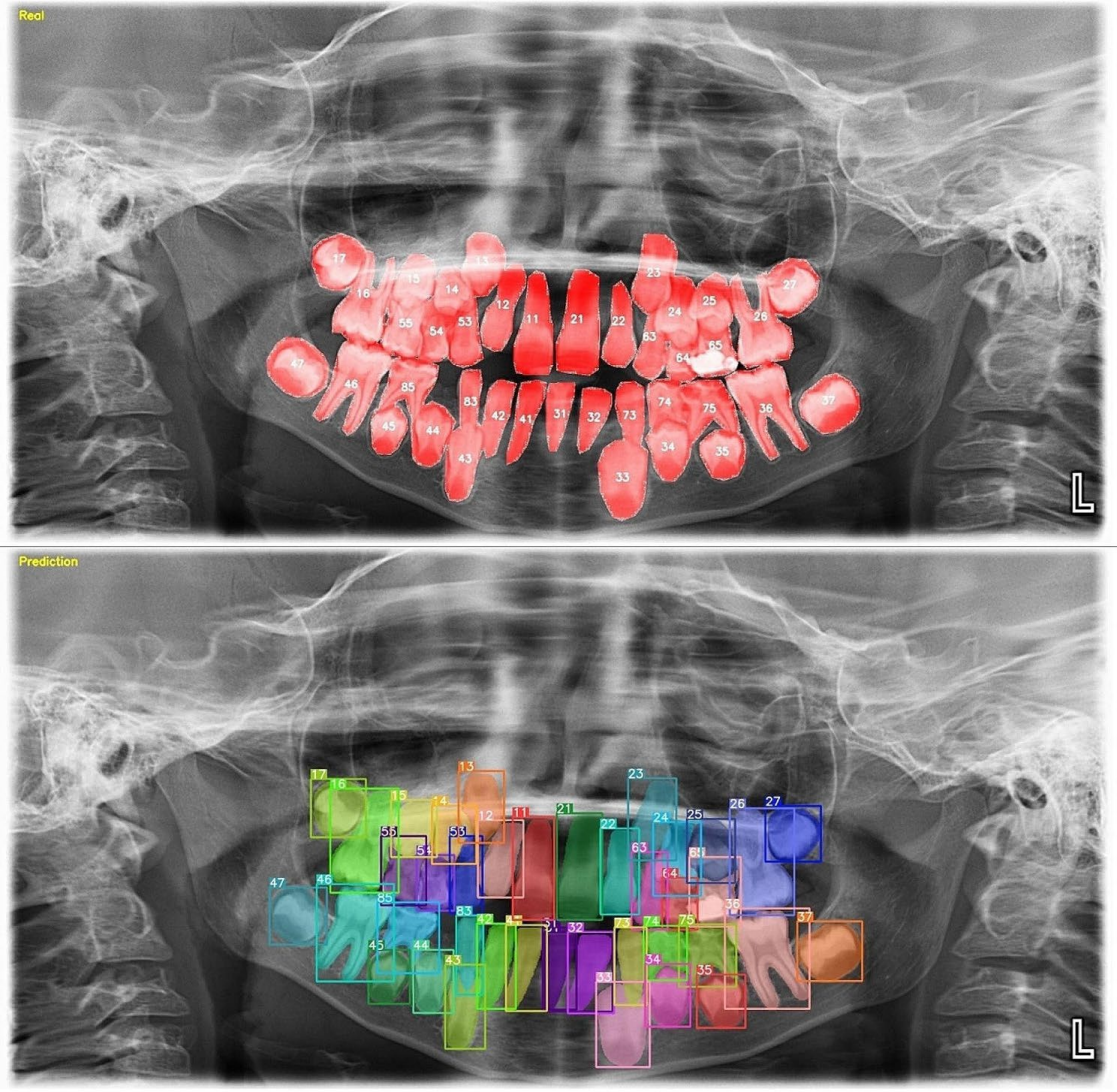
Tooth segmentation in PRs of pediatric patients with mixed dentition using an AI model

157,377 labels and a total of 3854 mixed images were made for both the detection task and the segmentation task. AI models based on the Deep-CNN architecture have shown near-perfect results for the detection and segmentation of deciduous and permanent teeth in PRs in mixed dentition pediatric patients (Figs. 3 and 4).

### Detection model

A manual count of the labels produced by the AI model used in the detection test phase showed that 15,519 labels were correct, 36 labels were incorrect, and 55 teeth were not detected. The sensitivity, precision, F-1 score, and mAP-0.5 values were 0.99, 0.99, 0.99, and 0.98 respectively, for tooth detection (Table 1).

**Table 1:** Predictive performance measurement using the AI model on test data (CranioCatch, Eskişehir, Turkey)

	Train (Images/Labels)	Validation (Images/Labels)	Test (Images/Labels)	True positive	False positive	False negative	Sensitivity	Precision	F1-Score	mAP-0.5
Detection Model	3093/125,743	387/16,060	385/15,574	15,519	36	55	0,99	0,99	0,99	0,98
Segmentation Model	3093/125,743	387/16,060	385/15,574	15,442	107	49	0,98	0,98	0,98	0,98

**Fig. 5:**
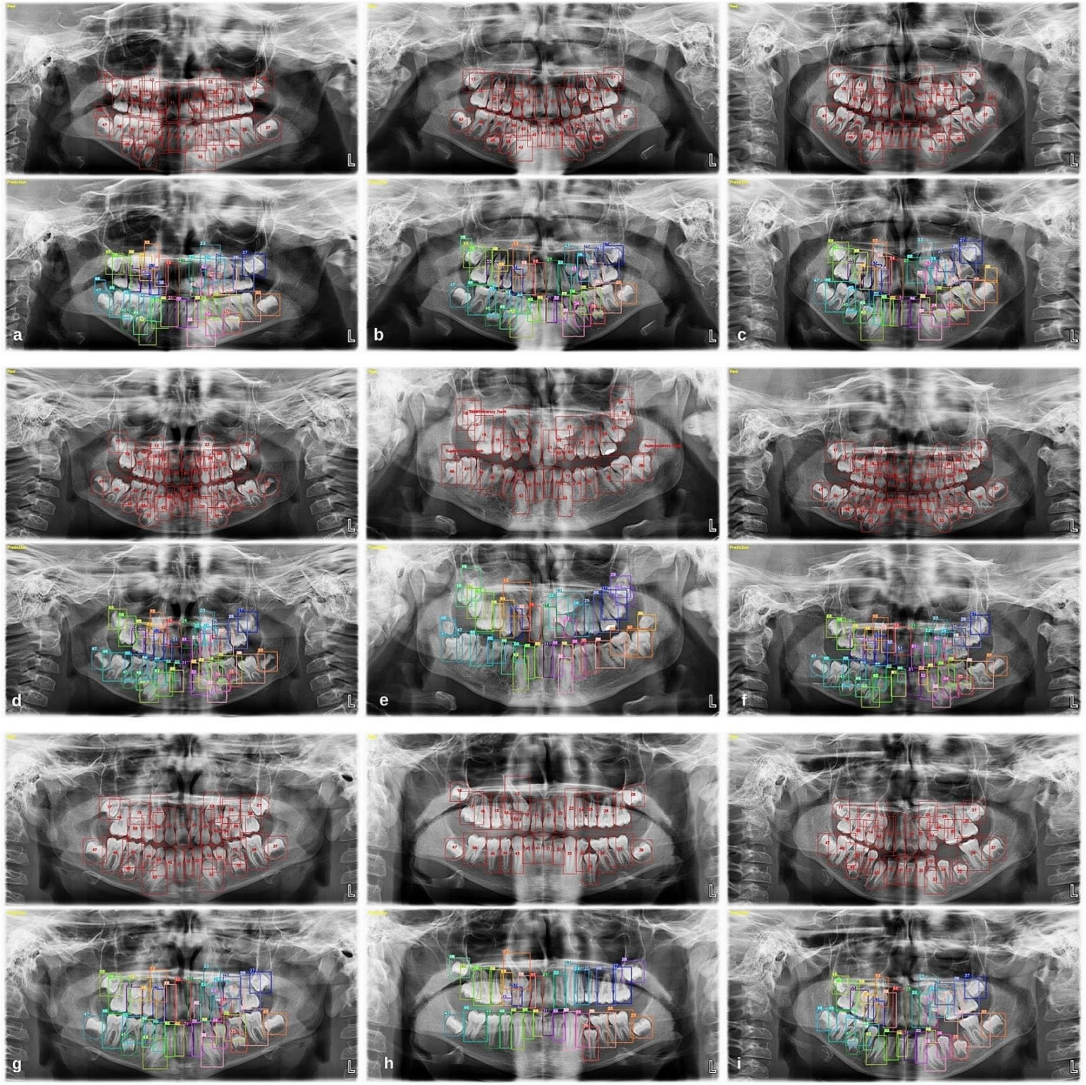
Examples of errors found in the detection model: (**a**) The model fails to detect when tooth germ is present, (**b**) The model detects when tooth germ is absent, (**c**) The model fails to detect when tooth morphology is distorted, (**d**) The model incorrectly detects when tooth morphology is distorted, (**e**) The model detects a supernumerary tooth as a normal tooth, (**f**) Inability to detect the existing tooth as a result of superposition of teeth in the anterior region, (**g**) Identification of the broken fragment as a tooth by the model as a result of tooth fracture, (**h**) Identifying caries in the crown as a tooth, (**i**) Impacted tooth 47 detected by the model as tooth 48

During the evaluation of errors in the detection model, it was found that the DL model created had the most difficulty detecting germs in wisdom teeth. Additionally, it struggled to identify teeth with damaged morphology caused by caries, trauma, or other factors. Mislabelling or failure to detect teeth in the anterior region was found to occur less frequently, but it was still a significant issue due to superimpositions in the PRs (Fig. 5).

### Segmentation model

With the objective of evaluate the segmentation success of the model, a threshold value of 0.50 was set for the Intersection over Unity (IoU) value. This threshold was selected as a predicted segmentation is considered correct if the IoU score exceeds or equals 0.50 with the ground truth. A manual count of the labels produced by the AI model used in the segmentation test phase showed that 15442 labels were correct, 107 labels were incorrect, and 49 teeth were not segmented. The sensitivity, precision, F-1 score, and mAP-0.5 values were 0.98, 0.98, 0.98, and 0.98, respectively, for tooth segmentation (Table 1).

**Fig. 6:**
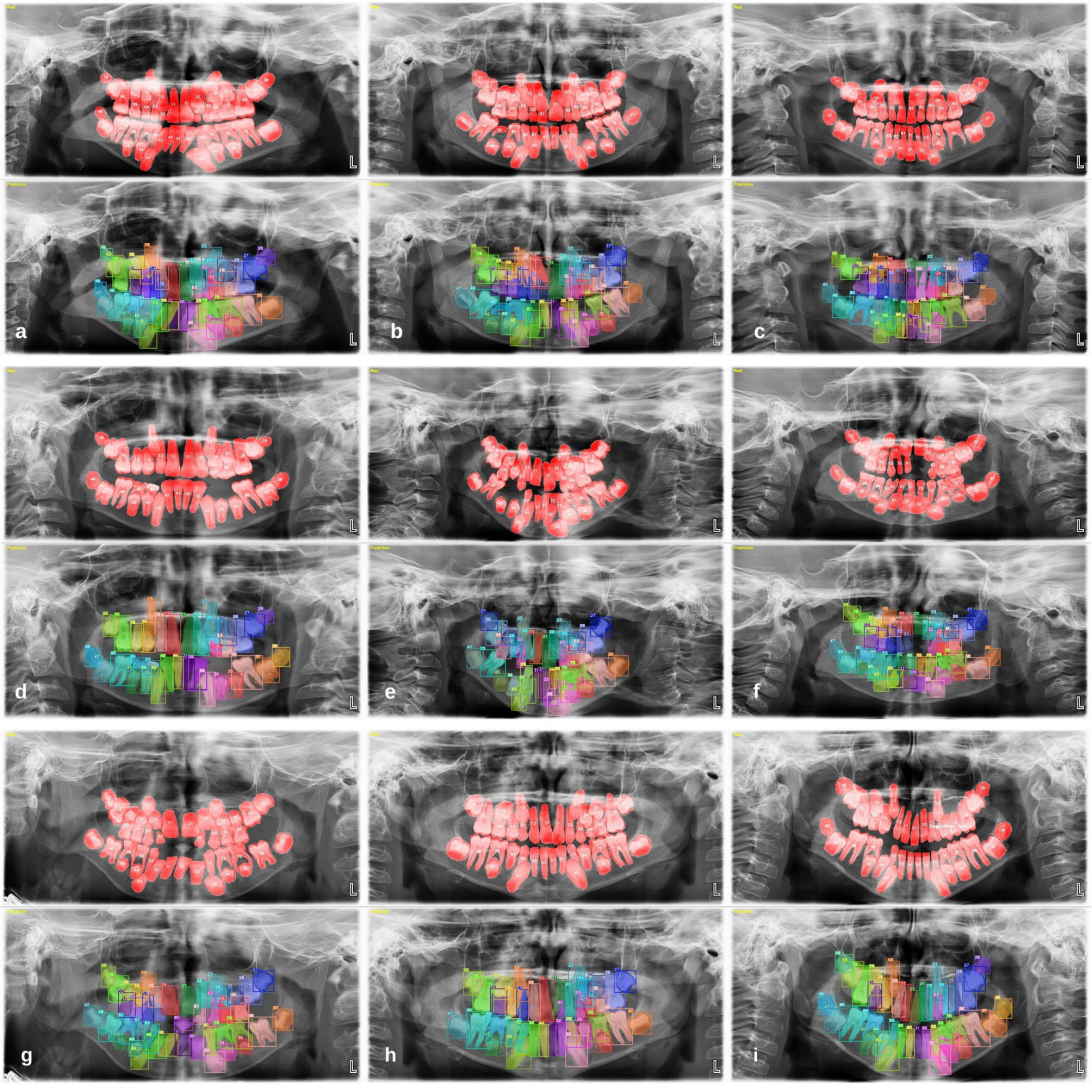
Examples of errors found in the segmentation model: (**a**) Double labelling during tooth segmentation in the posterior region due to superpositions caused by the coexistence of permanent tooth germs and deciduous teeth in the mixed dentition period, (**b**) Similarly, double labelling during tooth segmentation due to superpositions in the anterior region, (**c**) Segmenting the germ when the permanent tooth germ is not present, (**d**) Failing to segment this germ when the permanent tooth germ is present, (**e**) The model marks the numbers of the teeth in the right region as the number of the teeth in the left region, (**f**) Inability of the model to segment the teeth due to crowding in the lower anterior region, (**g**) Due to the lack of teeth, the model segmented a toothless area as a tooth, (**h**) the crown of tooth 21 was fractured and the model segmented the fractured area as tooth 61, (**i**) Segmentation and numbering of supernumerary teeth as normal teeth of the model

During the evaluation of errors in the segmentation model, it was found that double labelling occurred in the posterior and anterior regions due to the superposition of teeth. Furthermore, the model encountered challenges in accurately identifying the location of the teeth, resulting in mislabelling of the numbers of teeth on the left side as those on the right side and vice versa in certain radiographs. Occasionally, the model may encounter issues with crowding, missing teeth, or the loss of recognizable tooth features. Similarly, the segmentation model may incorrectly detect a germ or fail to detect an existing one, although this occurs less frequently than with the detection model (Fig. 6).

## Discussion

Although the head, neck, and cervical spine are in the field of view of many diagnostic images, teeth are rarely the primary focus of imaging studies. Therefore, radiographs dedicated primarily to the evaluation of the dentition may surprise many radiologists. Add to this the unusual tooth structure found in pediatric patients, and radiologists may be even more discouraged from confidently interpreting PRs [23]. Manual identification of teeth in dental radiographs is a time-consuming and error-prone process. Analysing these processes using deep learning can reduce the clinician’s workload and stress while minimizing the errors that may occur. As a result, many researchers are developing AI solutions based on DL [24]. Although there have been previous studies in the literature on tooth identification in PRs of pediatric patients during the mixed dentition period, no study using the YOLO-V5 deep learning model has been identified. In this study, we used various tooth identification methods, both segmentation and detection, by developing the YOLO-V5 DL model and applying it to PRs of a pediatric patient population with mixed dentition.

Numerous studies have been conducted on tooth detection and segmentation in PRs [19–21, 25–29], periapical radiographs [30–32], bite-wing radiographs [33–35], and Cone Beam Computed Tomography (CBCT) [36–38]. DL has been found to be successful in this regard. However, it is important to note that many of these studies have been conducted with small datasets and have focused on a single aspect of tooth identification and applied this method to permanent teeth [29]. In a meta-analysis of deep learning for tooth identification and numbering in dental radiographs, Sadr et al. reported that DL had an accuracy range of 81.8–99% and a precision range of 84.5–99.94% for tooth numbering and segmentation, according to all included studies. Furthermore, sensitivity was reported between 75.5% and 98%, specificity between 79.9% and 99%, precision between 82.7% and 98%, and F1-score between 87% and 98%. When PR was evaluated separately, the accuracy rate was reported to be 87.21–94.32% in studies using object detection and 93.2–99% in studies using classification. The sensitivity range of studies using periapical radiography varied between 91.4% and 96.1%, while the sensitivity range for studies using CBCT was reported to be between 93.8% and 98%.^24^

AI can also be used in pediatric dentistry. These models are extremely beneficial on an individual and social level, and they are excellent at categorizing children into risk categories. Furthermore, they can aid in the development of oral health programs in schools and raise children’s awareness of their dental health [39]. Additionally, DL models can assist in the examination of PRs in pediatric dentistry. In their deep learning study, Bağ et al., developed YOLO-v5 models to automatically detect nine important anatomical structures in approximately one thousand panoramic radiographs of pediatric patients. The F1 score and sensitivity values for the labelled anatomical regions were 0.98–0.99 for maxillary sinus, 1–1 for orbit, 0.97–0.99 for mandibular canal, 0.88–0.92 for mental foramen, 0.95–0.95 for foramen mandibula, 0.99–0.99 for incisura mandibula, 0.92–0.92 for articular eminence, 0.94–0.99 for condylar, and 0.86–0.97 for coronoid [7]. 

Ahn Y. et al., developed different DL models using SqueezeNet, ResNet-18, ResNet-101 and Inception-ResNet-V2 to detect mesiodens in PRs of primary or mixed dentition children. Accuracy, precision, recall and F1 scores were 0.95-0.96-0.90-0.93 for general dentists, 0.99-0.99-1.00-0.93 for pediatric specialists, 0.65-0.60-0.88-0.72 for SqueezeNet, 0.82-0.86-0.76-0.81 for ResNet-18, 0.86-0.85-0.88-0.86 for ResNet-101 and 0.88-0.87-0.90-0.88 for Inception-ResNet-V2. In their study, when the classification abilities of the DL models were compared with those of general dentists and pediatric dentists, it was observed that the accuracy of the DL models was lower than that of the dentists, but the detection was significantly faster. However, considering the success of their models in detecting mesiodens in panoramic radiographs, they stated that these models can help clinicians with limited clinical experience detect mesiodens [40]. Kim et al., developed a DL system using DeeplabV3 plus and Inception-resnet-v2 to identify mesiodens. The automatic segmentation method achieved high accuracy, precision, recall, F1-score, and area under the curve values for mesiodens diagnosis, all of which were 0.971 [41]. Mine et al., observed that DL models using AlexNet, VGG16-TL, and InceptionV3-TL all showed high performance in the classification and detection of supernumerary teeth in PRs. Accuracy, sensitivity, and specificity values for radiographs with single supernumerary teeth in the dataset were 79.5, 79.0 and 80.0 for Alex-Net, 84.0, 85.0 and 83.0 for VGG16-TL, 80.0, 82.0 and 78.0 for InceptionV3-TL. In radiographs with both single and double supernumerary teeth in the data set, these values were 80.5, 82.5 and 78.0 for AlexNet, 82.3, 85.0 and 79.0 for VGG16-TL, 80.9, 83.3 and 78.0 for InceptionV3-TL, respectively. As a result, they suggested that CNN-based DL is a promising approach for detecting of supernumerary teeth in the early mixed dentition stage [42]. Kaya et al., developed YOLO-v4 models for permanent tooth germ detection in PRs of pediatric patients. The YOLO-v4 model achieved a precision of 0.89, a recall of 0.91, and an F1-score of 0.90. The average precision value was calculated as 94.16% using the area under the sensitivity-recall curve [43]. Ha et al., succeeded in detecting mesiodens in PRs as a result of their model using YOLOv3. They showed that DL systems are effective in clinical practice to detect mesiodens in PRs of all dentition stages. The study evaluated model performance on 130 internal and 116 external images across three dentition groups: primary, mixed, and permanent dentition. The original images were preprocessed using contrast-limited histogram equalization (CLAHE) to investigate its effect. The results showed an accuracy of 96.2% for the internal test dataset and 89.8% for the external test dataset on the original images. The internal test dataset accuracy for primary, mixed, and permanent dentition was reported as 96.7%, 97.5%, and 93.3%, respectively. The external test dataset accuracy was reported as 86.7%, 95.3%, and 86.7%, respectively [44]. In addition, only one study was found on caries detection in PRs of pediatric patients. Zhang et al. reported good performance of the U-Net DL model they developed. The Recall, Specificity, Accuracy, IoU and Dice index results obtained as a result of 163 training images and 30 test images trained only on the child dental dataset are 0.92-0.98-0.97-0.83-0.91 for U-Net, 0.88-0.98-0.96-0.82-0.90 for R2 U-Net, 0.88-0.98-0.96-0.82-0.90 for PSPNet and 0.94-0.97-0.96-0.81-0.89 for Deeplab V3 + [45]. Kılıç et al., developed Faster R-CNN Inception v2 (COCO) models to automatically detect and number deciduous teeth in pediatric PRs. The models achieved a sensitivity of 0.98, precision of 0.95, and F1 score of 0.96 [20]. Zhu et al., found that the nnU-Net DL model was consistent and accurate in detecting and segmenting ectopic eruptions in mixed dentition molars. In their studies, the nnU-Net achieved an IoU of 0.834, precision of 0.845, F1-score of 0.902, and accuracy of 0.990. In comparison, the dentists achieved a mean IoU of 0.530, mean precision of 0.539, mean F1-score of 0.699, and mean accuracy of 0.811 [46]. Liu et al., developed an automated screening approach that can identify ectopic upper molar eruption with an accuracy comparable to that of pediatric dentists. The positive and negative predictive values of this automated screening system are 0.86 and 0.88, respectively, and its specificity and sensitivity are reported to be 0.90 and 0.86, respectively. They concluded that AI-based image recognition models can improve the accuracy of human interpreters but added that using DL for identification is still not 100% accurate [47].

In PRs of the pediatric population, there is limited research on tooth segmentation and detection using DL models. In their study, Pinherio et al. aimed to investigate tooth numbering and specimen segmentation tasks by creating a large PR dataset containing primary and permanent teeth. To this end, they used two different approaches based on Mask R-CNN, one using a traditional fully convolutional network (FCN) and the other integrating the PointRend module to improve the boundaries. The results show that + PointRend does better than + FCN at the sample segmentation and enumeration tasks. In particular, the improved boundary predictions made by + PointRend work much better for teeth that are big and have a lot of different shapes. They conclude that this study can provide an effective method for automated numbering and sampling of deciduous and permanent teeth in PRs [48]. Kaya et al., aimed to evaluate the performance of a DL system based on YOLO-V4 for automatic tooth detection and numbering in PRs of pediatric patients between the ages of 5 and 13, and obtained mAP value of 92.22%, an mean average recall (mAR) value of 94.44% and a weighted F1 score of 0.91. Accordingly, they reported that YOLO-V4 provides high and fast performance for automatic tooth detection and numbering in pediatric panoramic radiographs [49]. Bumann et al. developed a new collaborative Mask R-CNN based learning model that simultaneously identifies and discriminates between deciduous and permanent teeth and detects fillings. In their study, they created models that can identify deciduous and permanent teeth (mAP 95.32% and F1 score 92.50%) and their associated dental fillings (mAP 91.53% and F1 score 91.00%). They also designed a new method for collaborative learning using these two classifiers to improve the recognition performance and obtained 94.09% for mAP and 93.41% for F1 score [2]. Xu et al., developed a U-Net-based detection model and a ResNet-50-based tooth segmentation model for patients with primary, mixed, and permanent dentition in the dataset. They aimed for this model to be able to handle primary, mixed, and permanent dentition, maintain high accuracy in the presence of tooth number anomalies, dental diseases, conditions, restorations, prostheses, or appliances, and remain consistent across different dental imaging devices. They tested their DL models on a set of 1,209 PRs and found that they were accurate and reliable for tooth sample segmentation and tooth numbering (more than 97%). The IoU value between predictions and ground truth reached 92%. They demonstrated that the DL model they developed performed well on PRs across all three stages of dentition. They looked at the cases that didn’t match and found that out of the 39,099 teeth in the test set of 1,209 panoramic radiographs, 563 teeth could not be found or had IoU values less than 0.75, 325 teeth were given the wrong number, and 519 predictions were not teeth or had IoU values less than 0.75. They reported that this discrepancy can occur in missing teeth when adjacent teeth fill the gap, dental defects that occur when a large number of teeth are missing, crowding, superimposition, loss of distinctive morphological features of the teeth or the presence of tooth-like structures [29]. In our study, the included panoramic radiographs comprised carious teeth, teeth exhibiting extensive loss of material due to caries or trauma, and supernumerary teeth. The developed YOLO-v5 based DL model did not encounter any difficulty in the detection or segmentation of decayed teeth. However, it exhibited limitations in the detection and segmentation of radiographs where the recognisable features of the tooth were obscured. The sensitivity, precision, and F-1 scores were calculated as 0.99 for tooth detection and 0.98 for tooth segmentation. Given that not all the teeth in the panoramic radiographs included in the study were completely healthy, it can be concluded that these obtained values represent a more favourable result than those reported in other studies on tooth detection and segmentation.

DL techniques are used for tooth detection and segmentation, as well as the detection of various dental anomalies and diseases. While there are numerous AI studies on the evaluation of dental caries and periodontal conditions, there are only a few studies on other dental conditions [50]. Studies have been conducted with the aim of developing DL algorithms for the detection of teeth with vertical root fractures [51], the segmentation of taurodont tooth [52], and the detection of root resorption [53]. Root resorption is a serious condition that can lead to tooth extraction if not treated early. Root resorption may occur due to inflammation caused by bacterial infection, trauma, physical or chemical irritation, or rapid maxillary expansion [54, 55]. Consequently, AI-supported systems can be developed for the early detection and prevention of this condition. Fukuda et al. reported that the sensitivity value of the model they developed was 93% in their study, in which they developed a DetectNet-based DL algorithm that can detect vertical root fractures in panoramic radiography images [51]. Duman et al. reported that the sensitivity value of the U-Net-based model they developed to automatically segment taurodont teeth in panoramic radiographs was 86.50%.^52^ Tamura et al. developed an EfficientNet-based model that can detect root resorption in panoramic radiographs. They reported that the accuracy value of the model they developed was 71%.^53^ In addition to the YOLO-V5-based deep learning algorithm that detects and segments only the teeth developed in our study, the incorporation of dental conditions such as root resorption of teeth into these models in future studies will facilitate the creation of more comprehensive studies.

In comparison to previous deep learning techniques employed in related studies in the literature, the YOLO-V5 models developed during the current study obtained significantly better outcomes. Coşkun et al. reported that in their study on mass detection in mammograms, YOLO-V5 outperformed older versions [56]. Similarly, Yilmaz et al. conducted a comparison of two DL methods, Faster R-CNN and YOLO-V4, for tooth classification in PRs. The study aimed to determine which method was more accurate, efficient, and capable of detection. The YOLO-V4 method achieved an average precision of 99.90%, recall of 99.18%, and F1 score of 99.54%. The Faster R-CNN method achieved an average precision of 93.67%, recall of 90.79%, and F1 score of 92.21%. Accordingly, it has been stated that the YOLO-V4 method outperforms the Faster R-CNN method in terms of tooth prediction accuracy, detection speed, and ability to detect impacted and erupted third molars [25]. Our study’s use of PRs from a single centre and at identical settings for the model’s training is one of its limitations. Future studies should use PR images obtained from multiple radiography devices to ensure more reliable results.

In this study, we developed YOLO-v5 models to automatically detect deciduous and unerupted permanent teeth in approximately four thousand PRs of mixed dentition pediatric patients. The models demonstrated a high level of success in detecting these teeth, surpassing the results reported in the literature. The detection of these teeth during the mixed dentition period, where unerupted impacted teeth and deciduous teeth coexist on PRs, is crucial for the early detection of diseases and pathologies. Therefore, it is of great importance to identify and enumerate these structures in the first step in many respects. Automatic detection, segmentation, and numbering of these structures will aid in the clinical decision-making process, increase dentists’ awareness during examinations, and facilitate diagnosis and treatment while saving time.

### Electronic supplementary material

Below is the link to the electronic supplementary material.

Supplementary Material 1

## Data Availability

The datasets used and/or analysed during the current study are available from the corresponding author on reasonable request.

## Abbreviations

PRs: Panoramic radiographs
AI: Artificial Intelligence
DL: Deep Learning
CNN: Convolutional Neural Networks
YOLO: You Only Look Once
CLAIM: Checklist for Artificial Intelligence in Medical Imaging
STARD: Standards for the Reporting of Diagnostic Accuracy Studies
JSON: JavaScript Object Notation
IoU: Intersection over Union
SGD: Stochastic Gradient Descent
TP: True Positive
FP: False Positive
FN: False Negative
FCN: Fully Convolutional Network
CBCT: Cone Beam Computed Tomography
mAP-0.5: Mean Average Precision-0.5
mAR: Mean Average Recall
