# Correction: YOLO-V5 based deep learning approach for tooth detection and segmentation on pediatric panoramic radiographs in mixed dentition

**DOI:** 10.1186/s12880-024-01410-5

**Published:** 2024-08-28

**Authors:** Busra Beser, Tugba Reis, Merve Nur Berber, Edanur Topaloglu, Esra Gungor, Münevver Coruh Kılıc, Sacide Duman, Özer Çelik, Alican Kuran, Ibrahim Sevki Bayrakdar

**Affiliations:** 1 Department of Orthodontics, Faculty of Dentistry, Recep Tayyip Erdogan University, Rize, Turkey; 2 Pedodontics, Private Practice, Trabzon, Turkey; 3 Department of Oral and Maxillofacial Radiology, Faculty of Dentistry, Inonu University, Malatya, Turkey; 4 Department of Pedodontics, Faculty of Dentistry, Inonu University, Malatya, Turkey; 5 Department of Pedodontics, Faculty of Dentistry, Beykent University, Istanbul, Turkey; 6 Department of Mathematics-Computer, Faculty of Science, Eskisehir Osmangazi University, Eskisehir, Turkey; 7 Department of Oral and Maxillofacial Radiology, Faculty of Dentistry, Kocaeli University, İzmit, Kocaeli, 41190 Turkey; 8 Department of Oral and Maxillofacial Radiology, Faculty of Dentistry, Eskisehir Osmangazi University, Eskişehir, Turkey

Correction to: Beser et al. BMC Medical Imaging (2024) 24:172

10.1186/s12880-024-01338-w

The original article contained incomplete reference. Reference [24] reads:

Sadr S, Rokhshad R, Daghighi Y et al. Deep learning for tooth identification and numbering on dental radiography: A systematic review.

It should have read:

Sadr S, Rokhshad R, Daghighi Y, Golkar M, Tolooie Kheybari F, Gorjinejad F, et al. Deep learning for tooth identification and numbering on dental radiography: a systematic review and meta-analysis. Dentomaxillofac Radiol [Internet]. 2024;53(1):5–21. Available from: 10.1093/dmfr/twad001.

The Original Article has been corrected.

