# Supplementary material for: YOLO-V5 based deep learning approach for tooth detection and segmentation on pediatric panoramic radiographs in mixed dentition
Source: BMC Med Imaging. 2024 Jul 11;24:172. doi: 10.1186/s12880-024-01338-w (PMC11238494; doi:10.1186/s12880-024-01338-w)
Supplement: Supplementary file 1 — Supplementary Material 1 [file 12880_2024_1338_MOESM1_ESM.docx]

**Appendix A**

The Eskisehir Osmangazi University Faculty of Dentistry’s Dental Artificial Intelligence (AI) Laboratory has advanced technology computer equipment including a Dell PowerEdge T640 Calculation Server (Intel Xeon Gold 5218 2.3G, 16C/32T, 10.4GT/s, 22M Cache, Turbo, HT (125W) DDR4-2666, 32GB RDIMM, 3200MT/s, Dual Rank, PERC H330+ RAID Controller, 480GB SSD SATA Read Intensive 6Gbps 512 2.5in Hot-plug AG Drive); PowerEdge T640 GPU Calculation Server (Intel Xeon Gold 5218 2.3G, 16C/32T, 10.4GT/s, 22M Cache, Turbo, HT (125W) DDR4-2666 2, 32GB RDIMM, 3200MT/s, Dual Rank, PERC H330+ RAID Controller, 480GB SSD SATA Read Intensive 6Gbps 512 2.5in Hot-plug AG Drive, NVIDIA Tesla V100 16G Passive GPU); PowerEdge R540 Storage Server (Intel Xeon Silver 4208 2.1G, 8C/16T, 9.6GT/s, 11M Cache, Turbo, HT (85W) DDR4-2400, 16GB RDIMM, 3200MT/s, Dual Rank, PERC H730P+ RAID Controller, 2Gb NV Cache, Adapter, Low Profile, 8TB 7.2K RPM SATA 6Gbps 512e 3.5in Hot-plug Hard Drive, 240GB SSD SATA Mixed Use 6Gbps 512e 2.5in Hot plug, 3.5in HYB CARR S4610 Drive); Precision 3640 Tower CTO BASE Workstation (Intel(R) Xeon(R) W-1250P (6 Core, 12M cache, base 4.1GHz, up to 4.8GHz) DDR4-2666, 64GB DDR4 (4 X16GB) 2666MHz UDIMM ECC Memory, 256GB SSD SATA, Nvidia Quadro P620, 2GB); Dell EMC Network Switch (N1148T-ON, L2, 48 ports RJ45 1GbE, 4 ports SFP+ 10GbE, Stacking).
